# How politics affect pandemic forecasting: spatio-temporal early warning capabilities of different geo-social media topics in the context of state-level political leaning

**DOI:** 10.3389/fpubh.2025.1618347

**Published:** 2025-07-01

**Authors:** Dorian Arifi, Bernd Resch, Mauricio Santillana, Steffen Knoblauch, Sven Lautenbach, Thomas Jaenisch, Ivonne Morales

**Affiliations:** ^1^IT:U Interdisciplinary Transformation University Austria, Linz, Austria; ^2^Geoinformatics Department - Z_GIS, University of Salzburg, Salzburg, Austria; ^3^Center for Geographic Analysis, Harvard University, Cambridge, MA, United States; ^4^Machine Intelligence Group for the Betterment of Health and the Environment, Northeastern University, Boston, MA, United States; ^5^Harvard T. H. Chan School of Public Health, Department of Epidemiology, Boston, MA, United States; ^6^Klaus Tschira Stiftung, Heidelberg Institute for Geoinformation Technology, Heidelberg, Germany; ^7^Interdisciplinary Centre of Scientific Computing, Heidelberg University, Heidelberg, Germany; ^8^GIScience Chair, Heidelberg University, Heidelberg, Germany; ^9^Colorado School of Public Health, University of Colorado Boulder, Aurora, CO, United States; ^10^Department of Infectious Diseases, Heidelberg University Hospital, Heidelberg, Germany

**Keywords:** spatio-temporal semantic analysis, spatio-temporal epidemiology, geo-social media, political polarization, epidemiological early warning

## Abstract

**Objectives:**

Due to political polarization, adherence to public health measures varied across US states during the COVID-19 pandemic. Although social media posts have been shown effective in anticipating COVID-19 surges, the impact of political leaning on the effectiveness of different topics for early warning remains mostly unexplored. Our study examines the spatio-temporal early warning potential of different geo-social media topics across republican, democrat, and swing states.

**Methods:**

Using keyword filtering, we identified eight COVID-19-related geo-social media topics. We then utilized Chatterjee's rank correlation to assess their early warning capability for COVID-19 cases 7 to 42 days in advance across six infection waves. A mixed-effect model was used to evaluate the impact of timeframe and political leaning on the early warning capabilities of these topics.

**Results:**

Many topics exhibited significant spatial clustering over time, with quarantine and vaccination-related posts occurring in opposing spatial regimes in the second timeframe. We also found significant variation in the early warning capabilities of geo-social media topics over time and across political clusters. In detail, quarantine related geo-social media post were significantly less correlated to COVID-19 cases in republican states than in democrat states. Further, preventive measure and quarantine-related posts exhibited declining correlations to COVID-19 cases over time, while the correlations of vaccine and virus-related posts with COVID-19 infections.

**Conclusion:**

Our results highlight the need for a dynamic spatially targeted approach that accounts for both how regional geosocial media topics of interest change over time and the impact of local political ideology on their epidemiological early warning capabilities.

## 1 Introduction

COVID-19 was declared a pandemic on March 12th, 2020, by the World Health Organization (WHO). The disease posed a significant societal threat due to its high contagiousness and severe impact on those infected (1). However, reliably predicting the impact of COVID-19 waves was a major challenge for policymakers and health experts worldwide (2). Political tensions, particularly in the US, further complicated the situation by politicizing and polarizing public responses to the pandemic (3–7). In response, researchers sought to integrate diverse digital data sources, such as geo-social media data, to improve COVID-19 modeling and develop early warning systems that better captured the disease's transmission dynamics (8–10).

Geo-social media data, referring to microblogs on social networks with explicit geo-references, offers a valuable tool for local event detection (11, 12). Therefore, many studies have explored the potential of geo-social media data for enhancing early warning systems during the COVID-19 pandemic (13). For instance, Kogan et al. (10) used geo-social media data at the US state level to predict COVID-19 cases early in the pandemic, while Stolerman et al. (9) showcased its value on US county-level.

The strength of social media data, however, lies in its ability to provide semantic insights into public sentiment (14), behavioral trends (15), or reactions to societal events (16). Thus, researchers have used geo-social media data to analyze various aspects of the COVID-19 pandemic, including public sentiment (17), attitudes toward health measures (18), and general trends and topics of discussion (19, 20). In this regard, Hussain et al. found that geo-social media data closely aligned with nationwide surveys in the US and UK (18). Techniques commonly employed for analyzing this semantic dimension include keyword filtering (9, 21, 22) unsupervised statistical methods like Latent Dirichlet Allocation (LDA) (17, 19), and machine learning models like Bidirectional Encoder Representations from Transformers (BERT) (18, 20, 23). In this study, however, we rely on traditional keyword filtering to identify topics related to local COVID-19 infection rates. While the integration of semantic modeling to assess the early warning potential of various geo-social media topics constitutes a key innovation of our analysis, the keyword filtering itself is not the central contribution of this research. We further discuss possible advantages and shortcomings of this methodological choice in our limitations section.

Research also indicates that topics of interest can vary depending on the political leaning of a geo-social media user (24–26). Political leaning can also influence attitudes toward pharmaceutical (27) and non-pharmaceutical interventions, such as mask-wearing (4), social distancing, or personal COVID-19 risk perceptions (28). Accordingly, Kaashoek et al. suggest that political differences may even manifest in varying mortality rates across regions (29). These findings emphasize the need for epidemiological early warning models to consider local political leaning. Supporting this, Arifi et al. found strong variation in the early warning capabilities of geo-social media data across different political clusters and COVID-19 waves (22). This study extends their analysis by examining how the early warning capabilities of different geo-social media topics related to COVID-19 changed across US states and over time in the context of political leaning.

In summary, while numerous studies have analyzed the semantic content of geo-social media data in the context of COVID-19, the role of regional political beliefs and related social media topics, as well as how they shape the effectiveness of early warning models over time, has not yet been fully explored. This study seeks to address this gap by evaluating the spatio-temporal dynamics of geo-social media topics as early warning indicators across regions with differing political leanings. Therefore, we address the following research questions:

1. Which emerging **spatial patterns** can be observed in the early warning capabilities of **different geo-social media topics over time**?
2. To what degree do the **early warning capabilities** of geo-social media topics **depend on the timeframe** in which they are discussed or the **political leaning** of a given state?

## 2 Data and methods

### 2.1 Study area and timeframe

The spatial unit of analysis of our study is US state-level, while we specifically focused on the contiguous US to ensure sufficient data availability and facilitate a more meaningful analysis of spatial patterns, avoiding potential biases due to unconnected regions. Furthermore, we chose an analysis timeframe which covers the most prominent COVID-19 waves and periods, with and without vaccine accessibility. In particular, our analysis spans from the beginning of the COVID-19 pandemic in the US (February 28, 2020) to the end of the first major Omicron wave (April 27, 2022) (30).

### 2.2 Data

#### 2.2.1 COVID-19 case data

The official daily COVID-19 cases data, employed in this study, was acquired from the not-for-profit public data aggregator USAFacts (31). We transformed their cumulative data into daily incidence data and subsequently applied a 14-day moving average to account for possible reporting delays and differing update cycles across states.

#### 2.2.2 Geo-social media data

We collected 727 million geo-social media posts from the X (formerly Twitter) REST and Streaming API (Application Programming Interface) access points. Using the X Rest API we were able to collect posts in a 7 day sliding window, while the Streaming API access point allowed us to capture a continuous real-time data flow. We specifically filtered only for posts including a geo-location, which can be given by a polygon (e.g., city, state) or a point location depicted by a longitude-latitude pair. In either case, the geolocation can be manually set by the user or reflect the actual location of the device where the post was sent from. For the subsequent analysis steps, we only utilized geo-social media posts which had geometries completely within a US state, which left us with about 420 million posts. In addition, prior studies have shown that a substantial proportion of geo-tagged posts on X may originate from cross-posting on other platforms (>97%), such as Instagram or Foursquare (32), suggesting that the data used in our analysis may reflect user behavior across multiple social media platforms. Please note that the API access has been restricted by X and comparable data can no longer be collected through academic access. Future data collection efforts of a similar kind will need a commercial agreement with X.

Furthermore, we filtered the geo-social media posts using predefined keyword sets to identify topics relevant to COVID-19. These included eight specific topics, focusing on virus-related discussions (*Virus* and *Symptoms*), health authority positions (*Health Officials*), non-pharmaceutical interventions (*Testing, Preventive Measures, Quarantine*), and pharmaceutical interventions (*Vaccination*). A *COVID-19 Baseline* topic was added to provide a comparison of the early warning capabilities between a broader geo-social media topic and more specific subtopics. The keywords used to define these topics were primarily derived from prior research on COVID-19-related geo-social media content. Specifically, the topics *Virus, Symptoms, Testing, Health Officials, Preventive Measures*, and *Quarantine* were largely informed by the work of Chandrasekaran et al. and Xue et al., both of whom applied LDA (Latent Dirichlet Allocation) (33) to identify core themes in COVID-19-related geo-social media posts (19, 34). While their unsupervised topic modeling approaches often resulted in overlapping keywords across topics, we aimed to minimize such overlap by carefully selecting distinct keyword sets and excluding ambiguous terms not directly related to COVID-19. In addition, given the extensive focus in the literature on vaccine-related discussions in geo-social media (27, 34), we included a dedicated vaccination topic into our analysis. Lastly, medical experts contributed to the expansion of our keyword list to better capture disease-specific terminology and symptoms. Table 1 shows the exact keywords used for each topic.

**Table 1:** Keywords used for relevant Tweet extraction.

**Topics**	**Keywords**
Virus	COVID, corona, sarscov, sars-cov, epidemic, pandemic, influenza, virus, viral, infect, 2019-ncov, Delta variant, Omicron, H1N1, H3N2, Wuhan, transmission, super spread, incubation
Symptoms	fever, cough, shortness of breath, sore throat, headache, fatigue, body aches, loss of taste, loss of smell, no smell, no taste, nasal congestion, runny nose, respirator, symptom
Testing	PCR, antigen, rapid, test
Vaccination	vaccin, booster, Pfizer, Moderna, AstraZeneca, Johnson & Johnson, Cominarty, Janssen, mrna, vax, Biontech, jab
Preventive measures	mask, face covering, FFP2, N95, KN95, KF94, stay safe, flatten the curve, handwashing, wash your hands
Quarantine	quarantine, lockdown, social distancing, stay-at-home, isolat, social distance, keep distance
Health officials	health expert, Fauci, world health organization, CDC, centers for disease control, virologist, immunologist, supportive care, hospital, ventilat, clinic, intensive care unit, FDA
COVID-19 baseline	All the above keywords were used for this topic.

The keyword filtering was performed using lowercase keywords only, however, for reasons of readability names and hashtags are written with capital letters.

Overall, we found about 24.8 million geo-social media posts including at least one keyword related to COVID-19, while posts could contain several different keywords at once. We aggregated daily geo-social media posts by state and topic and normalized by the total number of posts per state on each day. In addition, we applied a 14-day rolling average to smooth out outliers. The subsequent analyses used these topic-specific ratios.

#### 2.2.3 US state-level political clusters

We classified US states as republican, democrat, or swing states based on the MIT county-level voting data for the 2020 election (35), which we aggregated to the state level. However, identifying swing states is a complex and not undisputed task in political science. Some authors rely on definitions from news agencies (36), which may be biased (37), while others use thoroughly defined criteria like bellwether status and competitiveness, which are in nature somewhat qualitative making them difficult to use (38).

**Figure 1:**
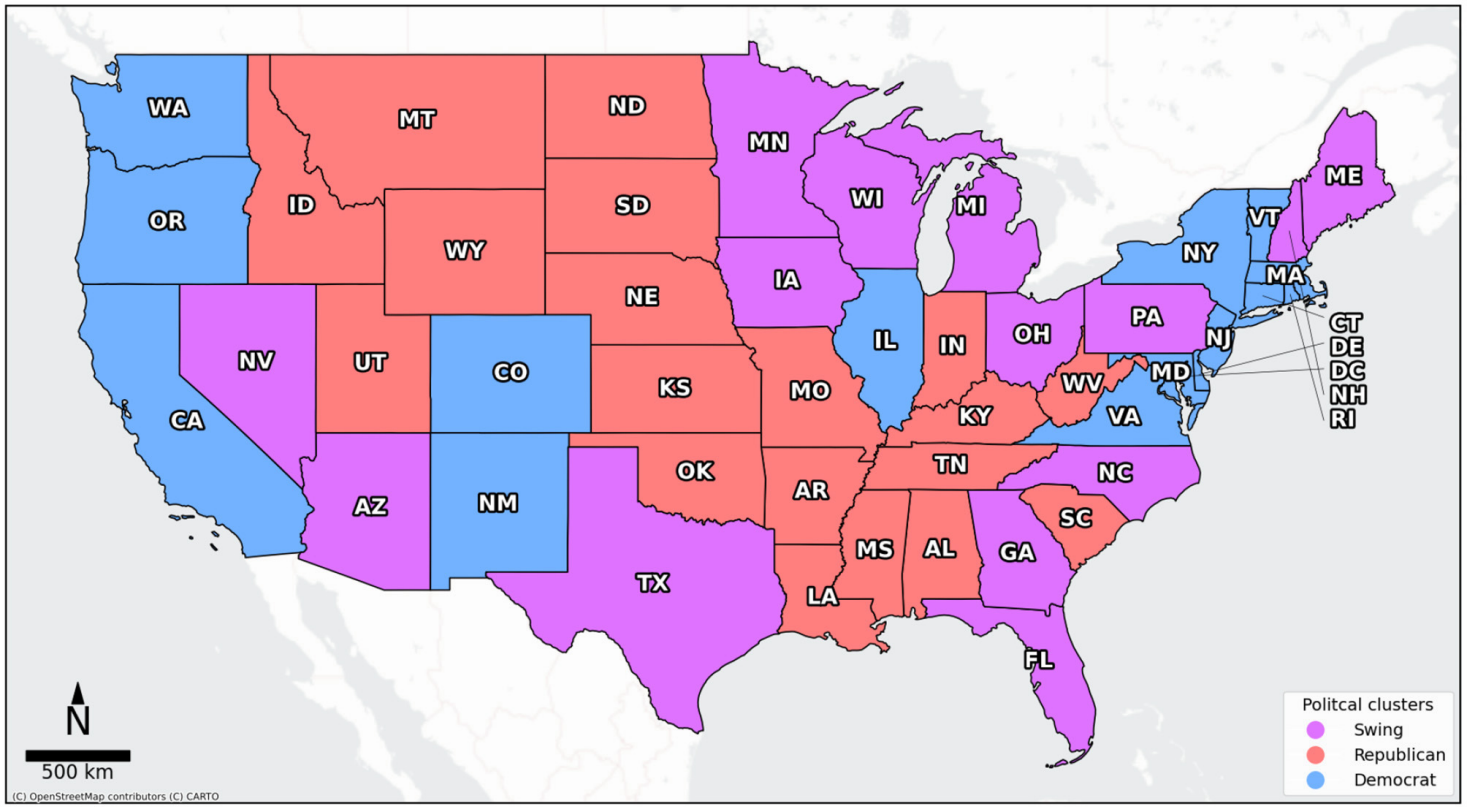
US state based political clusters. Please find a table with state abbreviations and the corresponding full state names in Table 3 in the Supplementary Appendix.

In this study, we sought to identify which geo-social media topics resonated most across regions with different political beliefs. Accordingly, we defined swing states as those where both parties exert a balanced influence, that is, where the difference between the republican and democrat vote share is < 10%, which is also in line with established political science conventions (38). Figure 1 shows a map of the resulting political clusters. Note, the source of all maps depicted in this study is © OpenStreetMap contributors © CARTO and the projections are Web Mercator (EPSG: 3857).

### 2.3 Methods

#### 2.3.1 Defining epidemiological waves

**Figure 2:**
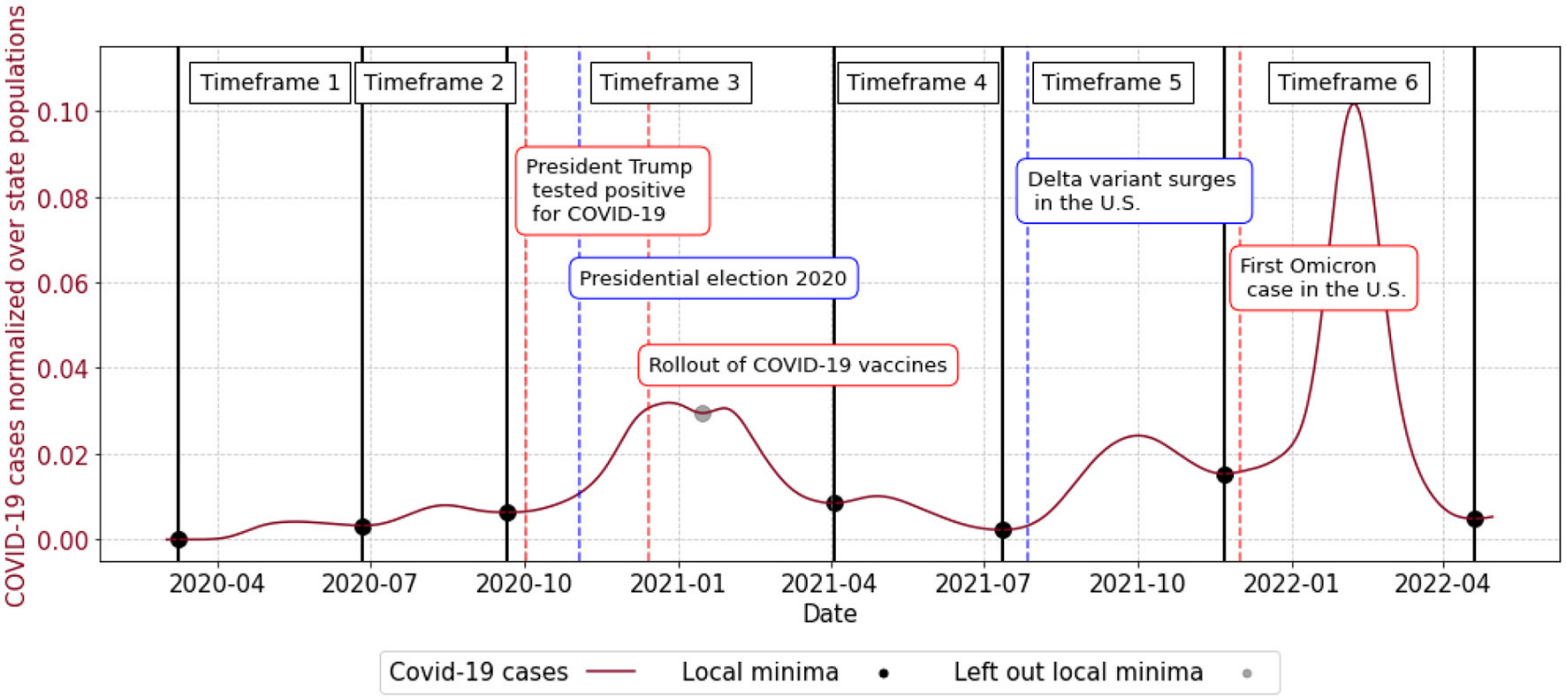
Timeframes capturing different waves of COVID-19 cases based on local minima.

To assess the early warning capabilities of geo-social media topics over time, we divided the US COVID-19 case time series into six epidemiological waves. In general, various methods exist to define such waves, using metrics like the effective reproduction number (29, 39), exponential growth models (10, 40), or data-driven thresholds (41). However, they all rely on subjective criteria to define an epidemiological wave. Thus, following the approach of Ayala et al. (41) and Arifi et al. (22), we used a data-driven approach, defining waves by splitting the 21-day moving average of US COVID-19 cases at their local minima (22, 41). Although this initially resulted in seven timeframes, we omitted the original third local minimum (January 2021) to avoid splitting the larger third wave (approximately ranging from October 2020 to April 2021) into separate phases. This left us with six distinct timeframes, which are together with additional information illustrated in Figure 2 (42, 43).

#### 2.3.2 Assessing early warning capabilities of geo-social media topics

We assessed the ability of geo-social media topics to provide early warning signals for COVID-19 cases within a 7 to 42 day window. This time window was based on prior results by Stolerman et al. (9), who found signals in digital traces anticipating COVID-19 cases up-to 6 weeks in advance (9). In detail, for each US state, we shifted the geo-social media time series forward by 7 to 42 days and computed Chatterjee's rank correlation with the COVID-19 case time series for each shift. This process was repeated for each topic across all epidemiological waves, with the topic achieving the highest correlation, at any shift, considered to have the strongest early warning capability. This is because Chatterjee's rank correlation quantifies the dependence between two sets of variables. Put differently, a high correlation value indicates that one set of values may be functionally related to and thus can be predictive of the other, suggesting a higher early warning capability. We disregarded correlations with Bonferroni-corrected *p-values* > 0.05 to account for multiple hypothesis.

## 3 Results

### 3.1 State-level spatial autocorrelation of geo-social media topics' early warning capabilities

**Figure 3:**
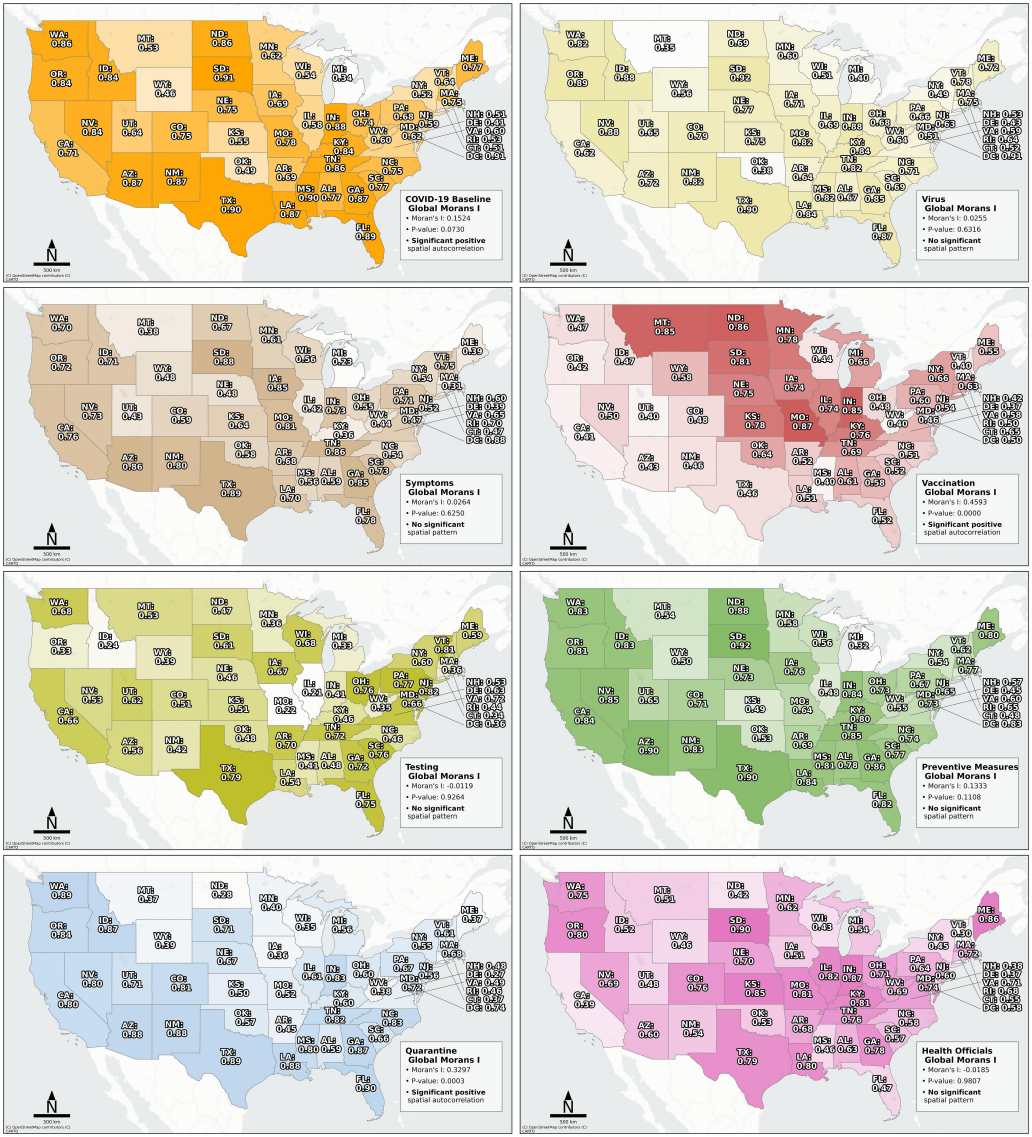
Chatterjee's rank correlation for each geo-social media topic for mainland US states in timeframe 2. Please find a table with state abbreviations and the corresponding full state names in Table 3 in the Supplementary Appendix.

Figure 3 illustrates the Chatterjee's rank correlation for each geo-social media topic to the COVID-19 cases across states and the emerging spatial patterns during timeframe 2. The eight different colors reflect the different topics. A global Moran's I analysis confirmed the significant positive spatial autocorrelations in the second timeframe for the *COVID-19 Baseline, Vaccination*, and *Quarantine* topic. Furthermore, the results clearly indicate that certain topics achieved higher correlations in specific spatial regimes. Notably, the *Vaccination* topic showed the highest correlations in the northern central states, while the *Quarantine* topic peaked in nearly opposite states in the southeast and west. A similar, albeit weaker, spatial opposition between these topics was observed during the presidential election in timeframe 3. Furthermore, the topics exhibiting positive spatial autocorrelation varied over the course of the pandemic and the corresponding maps for different timeframes can be found in Figures 6–10 in the Supplementary Appendix.

**Figure 4:**
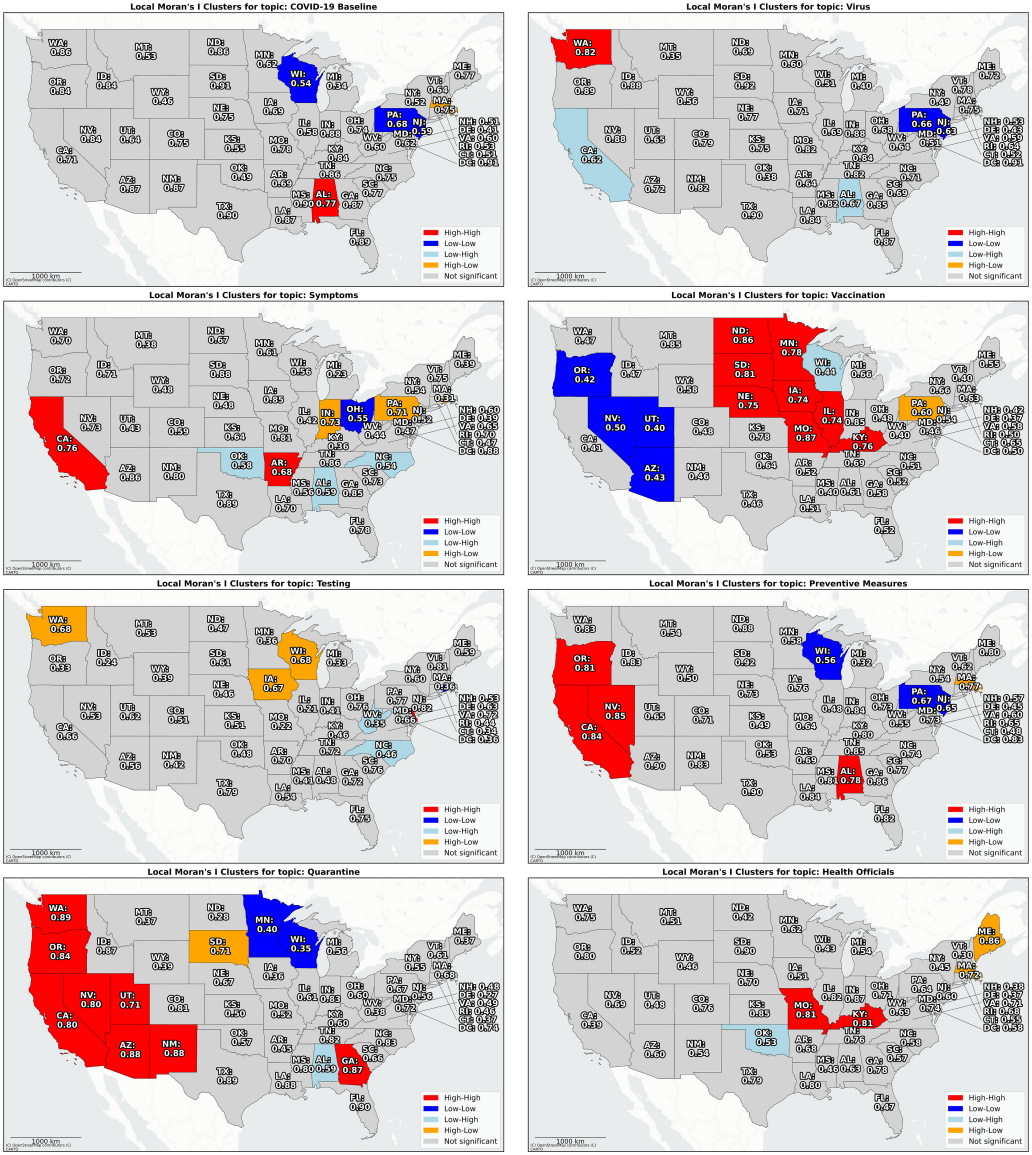
Local spatial autocorrelation over Chatterjee's rank correlation for each geo-social media topic for mainland US states in timeframe 2. Please find a table with state abbreviations and the corresponding full state names in Table 3 in the Supplementary Appendix.

Figure 4 resents Anselin's Local Moran's I for each topic during timeframe 2, using a queen contiguity spatial weights matrix. Consistent with the patterns shown in Figure 3, we observed a significant high-high cluster (hot spot) for the *Vaccination* topic in the mid-northern states, and a low-low cluster (cold spot) in the southwestern states. In contrast, the *Quarantine* topic reveals a significant hot spot in the southwestern region and a cold spot in the state of Minnesota. Additional maps depicting the local spatial autocorrelation for other topics are provided in the Supplementary Appendix (Figures 11–15).

### 3.2 Interaction effects of geo-social media topics with timeframe and political cluster

We utilized a linear mixed-effects model to assess the influence of the fixed effects *Topic, Timeframe*, and *Political_Cluster* on the *Correlation* between geo-social media posts and COVID-19 cases. Additionally, we introduced *Timeframe* as a random effect to control for variability in *Correlation* baselines across different timeframes. We introduced interaction effects between the variable *Topic* and *Timeframe* as well as between *Topic* and *Political_Cluster*. These interaction effects allowed to specifically test, whether the correlations between certain geo-social media topics and COVID-19 case varied depending on the political leaning of a state or the timeframe in which a topic was discussed. Note, we did not include an additional interaction effect between a state's political leaning and the time frame, as prior tests indicated that its coefficient was neither significant nor improved the model fit. To mitigate potential multicollinearity arising from keyword overlap between topic categories, we excluded the *COVID-19 Baseline* topic from the analysis. Although geo-social media posts can still be associated with multiple topic categories, diagnostic checks using Variance Inflation Factors (VIFs) indicated only moderate multicollinearity (VIFs < 10) for topic coefficients. While these levels of multicollinearity may still inflate standard errors, we deem them acceptable given the semantic complexity of social media data and the persistent significance of many topic coefficients. Further, to reduce heteroscedasticity in the models' residuals we disregarded samples exhibiting zero correlation. The model is depicted in Equation 1 and Equation 2.


(1) $$\begin{array}{l} Correlation=\beta_{0}+\beta_{1}\left(Timeframe\times Topic\right)\\ +\beta_{2}\left(Political\text{\_}Cluster\times Topic\right)+u_{Timeframe} \end{array}$$

(2) $$\begin{array}{l} \text{Where }u_{Timeframe}\sim N\left(0,\sigma_{Timeframe}^{2}\right) \end{array}$$

Table 2 shows the coefficients of the model in Equation 1. The results suggest that the geo-social media topics *Virus, Quarantine, Preventive Measures* achieved significantly higher influence on the correlation between geo-social media data and COVID-19 cases, compared to the *Health Officials* topics (reference category for *Topic*), while the *Testing* topic had a significantly lower influence. In addition, we found a significant negative interaction effect between the geo-social media topic *Quarantine* and *Political_Cluster*, indicating that the *Quarantine* topic is less effective for early warning in republican states than in democrat states (reference category for *Political*_*Cluster*), relative to the *Health Officials* topic (reference category for *Topic*). Beyond that, we also found significant interaction effects between the *Virus, Vaccination, Quarantine*, and *Preventive Measures* topics with the *Timeframe* variable, respectively. While the early warning capability of the *Preventive Measures* and the *Quarantine* topic declined over time, both the *Virus* and *Vaccination* topic showed an increasing trend over time. Furthermore, we employed a 10-fold cross-validation approach to assess the model's fit and found no significant differences across the MSE (mean: 0.6043), RMSE (mean: 0.7771), or MAE (mean: 0.6244) across all folds. The dependent variable was log-transformed to meet the normality assumptions of the residuals and ranged from −2.271 to 3.225.

**Table 2:** Coefficients of the linear mixed-effects model depicted in Equations 1, 2.

**Variable**	**Coefficient**	**Std. error**	***P-value***
*Intercept*	0.530	0.378	0.160
*Topic: Preventive_Measures*	0.483	0.122	0.000^***^
*Topic: Quarantine*	0.734	0.122	0.000^***^
*Topic: Symptoms*	0.102	0.122	0.402
*Topic: Testing*	−0.439	0.122	0.000^***^
*Topic: Vaccination*	−0.067	0.122	0.582
*Topic: Virus*	0.505	0.122	0.000^***^
*Political_Cluster: Republican*	0.055	0.090	0.539
*Political_Cluster: Swing*	0.041	0.097	0.671
*Topic: Preventive_Measures* × *Political_Cluster: Republican*	0.054	0.127	0.673
*Topic: Quarantine* × *Political_Cluster: Republican*	−0.243	0.127	0.055^*^
*Topic: Symptoms* × *Political_Cluster: Republican*	−0.115	0.127	0.367
*Topic: Testing* × *Political_Cluster: Republican*	−0.019	0.127	0.882
*Topic: Vaccination* × *Political_Cluster: Republican*	0.018	0.127	0.886
*Topic: Virus* × *Political_Cluster: Republican*	−0.040	0.127	0.753
*Topic: Preventive_Measures* × *Political_Cluster: Swing*	0.004	0.137	0.977
*Topic: Quarantine* × *Political_Cluster: Swing*	−0.031	0.137	0.819
*Topic: Symptoms* × *Political_Cluster: Swing*	−0.084	0.137	0.538
*Topic: Testing* × *Political_Cluster: Swing*	0.102	0.137	0.456
*Topic: Vaccination* × *Political_Cluster: Swing*	0.027	0.137	0.845
*Topic: Virus* × *Political_Cluster: Swing*	0.024	0.137	0.861
*Timeframe*	−0.122	0.123	0.324
*Topic: Preventive_Measures* × *Timeframe*	−0.085	0.031	0.007^***^
*Topic: Quarantine* × *Timeframe*	−0.065	0.031	0.037^**^
*Topic: Symptoms* × *Timeframe*	0.038	0.031	0.226
*Topic: Testing* × *Timeframe*	0.050	0.031	0.112
*Topic: Vaccination* × *Timeframe*	0.201	0.031	0.000^***^
*Topic: Virus* × *Timeframe*	0.087	0.031	0.005^***^
*Group Variable*	0.258	0.283	

× interaction effect between variable x and y.

*** *p* < 0.01;

** p < 0.05;

* p < 0.1.

**Figure 5:**
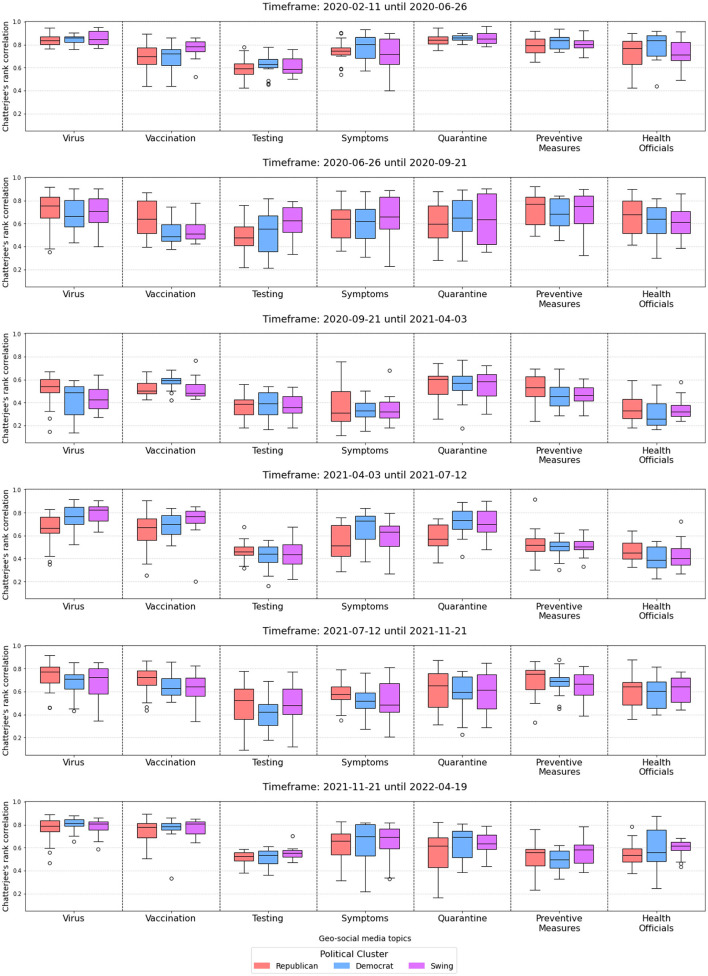
Chatterjee's rank correlation for each geo-social media topic per political cluster and per timeframe.

Furthermore, Figure 5 shows the corresponding distributions of Chatterjee's rank correlation between each geo-social media topic and COVID-19 cases time series, averaged over all states within the specified political cluster and for each timeframe.

## 4 Discussion

### 4.1 Principal results

Our findings validate and expand previous research highlighting the value of geo-social media data as an early warning tool for COVID-19 cases (9, 10, 20, 22, 44). We provide new insights into how the early warning capabilities of different geo-social media topics evolved across states, political beliefs, and over time. In detail, our results suggest that selecting geo-social media topics based on a dynamic spatially targeted approach, which accounts for different political ideologies and therewith associated differences in topics of interest, can improve the performance of future geo-social media-based early warning systems. However, it is important to highlight that our findings do not yet offer a straightforward approach to identifying the most promising geo-social media topic for epidemiological early warning in advance.

Spatial analysis revealed that the correlations of some geo-social media topics with COVID-19 cases appeared to be spatially clustered, while certain topics performed best in different and sometimes even opposing spatial regimes. This suggests that underlying spatial characteristics such as demographic, socio-economic, or political factors might have shaped the online discourse that reflects regional COVID-19 trends. In this regard, Jiang et al. (6) specifically point to the fact that the vast majority of hashtags related to the COVID-19 pandemic in the US were concerned with major political events or political leaders. This suggests that infection-related geo-social media discussions reflecting surges in COVID-19 cases, were most likely amplified by partisan political agendas. Accordingly, we found direct significant evidence that the early warning capability of geo-social media topics can depend on the political leaning of a state. Specifically, the early warning capability of the highly polarized *Quarantine* topic (28), was found to be weaker in republican compared to democrat states. This is also in line with the results by Arifi et al. (22), who found differences in the early waning capabilities for one broad geo-social media baseline topic across county-level political clusters. Our results expand on these findings by demonstrating that future epidemiological early warning systems can benefit from accounting for diverse regional geo-social media topics, particularly those reflecting the prevailing political leaning of a state.

Furthermore, we observed a significant decrease in the early warning capabilities of the geo-social media topic *Preventive Measures* and *Quarantine* over the course of the pandemic, whereas the topics *Vaccination* and *Virus* increased in effectiveness. This could indicate that the public interest in preventive measures like masks and hand washing to combat rising infections as well as quarantine measures, decreased over time and might have been overtaken by the emerging topics concerned with new virus variants and the increasingly polarized discourse surrounding the introduction of vaccines as a means to contain the virus (5, 7). These findings are also somewhat in line with results by Arifi et al. (22), who found a decreasing number of COVID-19 related posts over the course of the pandemic which they suggested might be caused by some kind of pandemic and/or social media fatigue that might have reduced online engagement with COVID-19. In contrast, our results, suggest that their observed decrease in posts, might reflect a shift in public interest, with attention moving from a static baseline topic with limited keywords toward emerging and at times polarizing new topics. This further emphasizes the need for a spatially targeted approach that can account for newly emerging local topics of discussion related to COVID-19 infections. However, it remains the task of future research to explore such approaches and to further examine how polarization of a topic might influence its epidemiological early warning capabilities.

### 4.2 Limitations

Chatterjee's rank correlation can identify whether a relationship between geo-social media posts and COVID-19 cases exists, however, it does not reveal their exact functional nature. Thus, it remains the task of future research to identify these functional relationships to build accurate prediction models. However, our study focused on assessing how different geo-social media topics impact epidemiological early warning, in the context of political leaning. The significant differences across observed across states, timeframes and topics underscore the spatial nature of this early warning capability, though further research is needed to confirm whether these patterns persist in more advanced prediction models.

We opted for a keyword filtering approach for the semantic analysis in parts due to the size of our dataset (>500 GB) and the high storage and computing resources machine learning methods would have demanded. In addition, we tested algorithms like for instance BERTopic (45) or LDA (33) on our data and different data subsets. However, these experiments yielded poor topic coherence, low precision, or failed due to resource constraints, even on GPU enabled compute clusters. In contrast, keyword filtering allowed for more precise topic definitions, while reducing topic overlap issues commonly observable in machine learning approaches (27). Moreover, our focus was not on advancing NLP techniques for large datasets but rather on examining the relationship between political leaning and geo-social media posts concerned with different discussion for epidemiological early warning, which keyword filtering effectively enabled. Nevertheless, future advances in machine learning may allow more sophisticated semantic analysis solutions, suitable for real-time application.

In addition, we acknowledge that our selected keywords may not fully capture all topics that were relevant throughout the pandemic. However, in defining the eight topics, we aimed to strike a balance between thematic relevance and analytical clarity. We deliberately excluded keywords that were difficult to assign to a specific topic [e.g., “panic buying” (19)] or a which were a priori highly politicized or predominantly used by one party [e.g., “small businesses, China” (24)]. This approach aimed to minimize bias in our comparison across states with different political leanings, ensuring that differences in early warning capability were not merely artifacts of topic polarization. Nevertheless, our findings still revealed significant differences in topic early warning capability across states with different political leaning, underscoring how deeply political dynamics shape regional public discourse and as a result the early warning capability of geo-social media data. Nevertheless, we recognize that future research could explore additional topics to further substantiate our findings.

Another limitation stems from the fact that our dataset only contains social media posts with an explicit geolocation. While this is vital for our analysis, studies suggest that only 0.85% of all posts on X included a geolocation (46) which introduces possible representation biases. Therefore, future work could utilize methods to infer geographic locations from the textual content of social media posts (e.g., named locations) without an explicit geolocation, as for instance introduced by Serere et al. (32), which may enhance spatial coverage and representativeness of the utilized data.

Further, our definition of timeframes is not without its difficulties and can influence the observed results. Specifically, state-specific factors such as holidays, lockdowns, and infection patterns exhibit differences across states, which inevitably influence the early warning capabilities of different geo-social media topics. Nevertheless, defining analysis timeframes based on the aggregate of COVID-19 cases over all states ensured comparability across states. Clearly future epidemiological analyses will not have the privilege of relying on timeframes defined on retrospective knowledge and will need to substantiate our findings in different infectious real-time early warning settings.

We also acknowledge that our results are most likely driven by underlying socio-economic conditions which constitute political beliefs. We tried to identify possible alternative explanatory variables instead of political beliefs to further understand the underlying driving factors for the differences in early warning capability across regions. Specifically, we included education level (share of college graduates) and population density, which are commonly associated with voting behavior (47, 48), as well as vaccination rates, which we used as a proxy for adherence to public health measures. While we did not observe significant coefficients for education and population density, we did observe a negative significant coefficient for vaccination rate as well as positive significant interaction effects between vaccination rate and the *Virus, Preventive Measures, Quarantine, Testing* and *Vaccination* topics (see Supplementary Appendix Table 4 for more details). Hence, it appears that the early warning potential of these topics improved in states where vaccination rates were rising. However, the precise dynamics driving the observed effects could not be conclusively determined within the scope of this study. Nonetheless, gaining insight into the underlying mechanisms behind the variation in early warning performance of different topics across politically distinct regions would significantly enhance future epidemiological models.

Lastly, this research explores the early warning capabilities of different geo-social media topics discussed on the platform X during the COVID-19 pandemic in the US, which might not be directly transferable to future epidemiological crises across different geographies. Also changes in executive company structure (49) or recommendation algorithm design (50) of geo-social media companies might lead to differing levels of polarization in future crises. In this regard, our research highlights how the early warning capabilities of different geo-social media topics can indeed depend on geographies, political beliefs and timeframe. Nevertheless, it remains a task of future research to assess to what degree the here presented results hold true for upcoming health crises, across different geographies and social media environments.

## Data Availability

The raw data supporting the conclusions of this article will be made available by the authors, without undue reservation.

## References

[1] Ciotti M Ciccozzi M Terrinoni A Jiang WC Wang CB Bernardini S. The COVID-19 pandemic. Crit Rev Clin Lab Sci. (2020) 57:365–88. 10.1080/10408363.2020.1783198 32645276

[2] Rashed EA Kodera S Hirata A. COVID-19 forecasting using new viral variants and vaccination effectiveness models. Comput Biol Med. (2022) 149:105986. 10.1016/j.compbiomed.2022.105986 36030722 PMC9381972

[3] Hart PS Chinn S Soroka S. Politicization and polarization in COVID-19 news coverage. Sci Commun. (2020) 42:679–97. 10.1177/1075547020950735 38602988 PMC7447862

[4] Kahane LH. Politicizing the mask: political, economic and demographic factors affecting mask wearing behavior in the USA. Eastern Econ J. (2021) 47:163–83. 10.1057/s41302-020-00186-0 33424048 PMC7783295

[5] Cowan SK Mark N Reich JA. COVID-19 vaccine hesitancy is the new terrain for political division among Americans. Socius. (2021) 7:23780231211023657. 10.1177/23780231211023657

[6] Jiang J Chen E Yan S Lerman K Ferrara E. Political polarization drives online conversations about COVID-19 in the United States. Hum Behav Emerg Technol. (2020) 2:200–11. 10.1002/hbe2.202 32838229 PMC7323338

[7] Tyson A Johnson C Funk C. US Public Now Divided Over Whether To Get COVID-19 Vaccine. Pew Research Center (2020). Available online at: https://www.pewresearch.org/science/2020/09/17/u-s-public-now-divided-over-whether-to-getcovid-19-vaccine/ (Accessed January 21, 2025).

[8] Liu D Clemente L Poirier C Ding X Chinazzi M Davis J. Real-time forecasting of the COVID-19 outbreak in Chinese Provinces: machine learning approach using novel digital data and estimates from mechanistic models. J Med Internet Res. (2020) 22:e20285. 10.2196/20285 32730217 PMC7459435

[9] Stolerman LM Clemente L Poirier C Parag KV Majumder A Masyn S. Using digital traces to build prospective and real-time county-level early warning systems to anticipate COVID-19 outbreaks in the United States. Sci Adv. (2023) 9:eabq0199. 10.1126/sciadv.abq0199 36652520 PMC9848273

[10] Kogan NE Clemente L Liautaud P Kaashoek J Link NB Nguyen AT. An early warning approach to monitor COVID-19 activity with multiple digital traces in near real time. Sci. Adv. (2021) 7:eabd6989. 10.1126/sciadv.abd6989 33674304 PMC7935356

[11] Havas C Resch B. Portability of semantic and spatial–temporal machine learning methods to analyse social media for near-real-time disaster monitoring. Nat Hazards. (2021) 108:2939–69. 10.1007/s11069-021-04808-4 34789962 PMC8550645

[12] Xu S Li S Huang W. A spatial-temporal-semantic approach for detecting local events using geo-social media data. Trans. GIS. (2020) 24:142–73. 10.1111/tgis.12589

[13] Tsao SF Chen H Tisseverasinghe T Yang Y Li L Butt ZA. What social media told us in the time of COVID-19: a scoping review. Lancet Digital Health. (2021) 3:e175–94. 10.1016/S2589-7500(20)30315-0 33518503 PMC7906737

[14] Ma N Yu G Jin X Zhu X. Quantified multidimensional public sentiment characteristics on social media for public opinion management: Evidence from the COVID-19 pandemic. Front Public Health. (2023) 11:1097796. 10.3389/fpubh.2023.1097796 37006559 PMC10060635

[15] Gu M Guo H Zhuang J Du Y Qian L. Social media user behavior and emotions during crisis events. Int J Environ Res Public Health. (2022) 19:5197. 10.3390/ijerph19095197 35564591 PMC9100990

[16] Abdukhamidov E Juraev F Abuhamad M El-Sappagh S AbuHmed T. Sentiment analysis of users' reactions on social media during the pandemic. Electronics. (2022) 11:1648. 10.3390/electronics11101648

[17] Boon-Itt S Skunkan Y. Public perception of the COVID-19 pandemic on Twitter: sentiment analysis and topic modeling study. JMIR Public Health Surveill. (2020) 6:e21978. 10.2196/21978 33108310 PMC7661106

[18] Hussain A Tahir A Hussain Z Sheikh Z Gogate M Dashtipour K. Artificial intelligence–enabled analysis of public attitudes on Facebook and Twitter toward COVID-19 vaccines in the United Kingdom and the United States: observational study. J Med Internet Res. (2021) 23:e26627. 10.2196/26627 33724919 PMC8023383

[19] Chandrasekaran R Mehta V Valkunde T Moustakas E. Topics, trends, and sentiments of Tweets about the COVID-19 pandemic: temporal infoveillance study. J Med Internet Res. (2020) 22:e22624. 10.2196/22624 33006937 PMC7588259

[20] Hanny D Arifi D Knoblauch S Resch B Lautenbach S Zipf A. An explainable GeoAI approach for the multimodal analysis of urban human dynamics: a case study for the COVID-19 pandemic in Rio de Janeiro. Comput Urban Sci. (2025) 5:13. 10.1007/s43762-025-00172-2 40046777 PMC11876275

[21] Jiang J Ren X Ferrara E. Social media polarization and echo chambers in the context of COVID-19: case study. JMIRx Med. (2021) 2:e29570. 10.2196/29570 34459833 PMC8371575

[22] Arifi D Resch B Santillana M Guan WW Knoblauch S Lautenbach S. Geosocial media's early warning capabilities across US county-level political clusters: observational study. JMIR Infodemiol. (2025) 5:e58539. 10.2196/58539 39883923 PMC11826950

[23] Salmi S Mérelle S Gilissen R van der Mei R Bhulai S. Detecting changes in help seeker conversations on a suicide prevention helpline during the COVID−19 pandemic: in-depth analysis using encoder representations from transformers. BMC Public Health. (2022) 22:530. 10.1186/s12889-022-12926-2 35300638 PMC8930480

[24] Guntuku SC Purtle J Meisel ZF Merchant RM Agarwal A. Partisan differences in Twitter language among US legislators during the COVID-19 pandemic: cross-sectional study. J Med Internet Res. (2021) 23:e27300. 10.2196/27300 33939620 PMC8176946

[25] Jing E Ahn YY. Characterizing partisan political narrative frameworks about COVID-19 on Twitter. EPJ Data Sci. (2021) 10:53. 10.1140/epjds/s13688-021-00308-4 34745825 PMC8556838

[26] Sylwester K Purver M. Twitter language use reflects psychological differences between democrats and republicans. PLoS ONE. (2015) 10:e0137422. 10.1371/journal.pone.0137422 26375581 PMC4574198

[27] Lyu H Wang J Wu W Duong V Zhang X Dye TD. Social media study of public opinions on potential COVID-19 vaccines: informing dissent, disparities, and dissemination. Intell. Med. (2022) 2:1–12. 10.1016/j.imed.2021.08.001 34457371 PMC8384764

[28] Allcott H Boxell L Conway J Gentzkow M Thaler M Yang D. Polarization and public health: Partisan differences in social distancing during the coronavirus pandemic. J Public Econ. (2020) 191:104254. 10.1016/j.jpubeco.2020.104254 32836504 PMC7409721

[29] Kaashoek J Testa C Chen JT Stolerman LM Krieger N Hanage WP. The evolving roles of US political partisanship and social vulnerability in the COVID-19 pandemic from February 2020–February 2021. PLoS Global Public Health. (2022) 2:e0000557. 10.1371/journal.pgph.0000557 36962752 PMC10021880

[30] Das S Samanta S Banerjee J Pal A Giri B Kar SS. Is Omicron the end of pandemic or start of a new innings? Travel Med Infect Dis. (2022) 48:102332. 10.1016/j.tmaid.2022.102332 35472451 PMC9033632

[31] USAFacts. US COVID-19 cases and deaths by state. (2020). Available online at: https://usafacts.org/visualizations/coronavirus-covid-19-spread-map/ (Accessed March 19, 2024).

[32] Serere HN Resch B Havas CR. Enhanced geocoding precision for location inference of tweet text using spaCy, Nominatim and Google Maps. A comparative analysis of the influence of data selection. PLoS ONE. (2023) 18:e0282942. 10.1371/journal.pone.0282942 36921000 PMC10016707

[33] Blei DM Ng AY Jordan MI. Latent dirichlet allocation. J Mach Learn Res. (2003) 3:993–1022.

[34] Xue J Chen J Hu R Chen C Zheng C Su Y. Twitter discussions and emotions about the COVID-19 pandemic: machine learning approach. J Med Internet Res. (2020) 22:e20550. 10.2196/20550 33119535 PMC7690968

[35] MIT Election Data and Science Lab. County Presidential Election Returns 2000–2020. (2018). Available online at: https://dataverse.harvard.edu/citation?persistentId= (Accessed March 8, 2024).

[36] Baccini L Brodeur A Weymouth S. The COVID-19 pandemic and the 2020 US presidential election. J Popul Econ. (2021) 34:739–67. 10.1007/s00148-020-00820-3 33469244 PMC7809554

[37] Abbas AH. Politicizing the pandemic: a schemata analysis of COVID-19 News in two selected newspapers. Int J Semiot Law. (2022) 35:883–902. 10.1007/s11196-020-09745-2 33214736 PMC7332744

[38] Schultz DA Jacob R Melcher JP Fried A. Presidential Swing States. Lanham: Lexington Books (2018). Available online at: https://scholarworks.umf.maine.edu/publications/99

[39] Zhang SX Arroyo Marioli F Gao R Wang S. A second wave? What do people mean by COVID waves? – A working definition of epidemic waves. Risk Manag Healthc Policy. (2021) 14:3775–82. 10.2147/RMHP.S326051 34548826 PMC8448159

[40] Kriston L. A statistical definition of epidemic waves. Epidemiologia. (2023) 4:267–75. 10.3390/epidemiologia4030027 37489498 PMC10366927

[41] Ayala A Dintrans PV Elorrieta F Castillo C Vargas C Maddaleno M. Identification of COVID-19 waves: considerations for research and policy. Int. J. Environ. Res. Public Health. (2021) 18. 10.3390/ijerph182111058 34769577 PMC8583384

[42] CDC. Centers for Disease Control and Prevention. CDC Museum COVID-19 Timeline (2023). Available online at: https://www.cdc.gov/museum/timeline/covid19.html (Accessed May 17, 2024).

[43] WHO. Classification of Omicron (B.1.1.529): SARS-CoV-2 Variant of Concern. (2021). Available online at: ttps://www.who.int/news/item/26-11-2021-classificationof-omicron-(b.1.1.529)-sars-cov-2-variant-of-concern (Accessed May 17, 2024).

[44] Comito C. How COVID-19 information spread in U.S.? The role of Twitter as early indicator of epidemics. IEEE Trans Serv Comput. (2022) 15:1193–205. 10.1109/TSC.2021.3091281

[45] Grootendorst M. BERTopic: neural topic modeling with a class-based TF-IDF procedure. (2022). Available online at: https://arxiv.org/abs/2203.05794 (Accessed February 24, 2024).

[46] Sloan L Morgan J. Who tweets with their location? Understanding the relationship between demographic characteristics and the use of geoservices and geotagging on Twitter. PLoS ONE. (2015) 10:e0142209. 10.1371/journal.pone.0142209 26544601 PMC4636345

[47] Brown TE Mettler S. Sequential polarization: the development of the rural-urban political divide, 1976–2020. Perspect Politics. (2024) 22:630–58. 10.1017/S1537592723002918

[48] Dawes CT Okbay A Oskarsson S Rustichini A. A polygenic score for educational attainment partially predicts voter turnout. Proc Nat Acad Sci. (2021) 118:e2022715118. 10.1073/pnas.2022715118 34873032 PMC8685665

[49] Schmidt S Zorenböhmer C Arifi D Resch B. Polarity-based sentiment analysis of georeferenced tweets related to the 2022 Twitter acquisition. Information. (2023) 14:71. 10.3390/info14020071

[50] Bellina A Castellano C Pineau P Iannelli G De Marzo G. Effect of collaborative-filtering-based recommendation algorithms on opinion polarization. Phys Rev E. (2023) 108:054304. 10.1103/PhysRevE.108.054304 38115540

