# Supplementary material for: How politics affect pandemic forecasting: spatio-temporal early warning capabilities of different geo-social media topics in the context of state-level political leaning
Source: Front Public Health. 2025 Jul 1;13:1618347. doi: 10.3389/fpubh.2025.1618347 (PMC12259613; doi:10.3389/fpubh.2025.1618347)
Supplement: Supplementary file 1 [file Data_Sheet_1.docx]

## Appendix

Table 3 Abbreviations and full names for US mainland states and Washington DC.

| # | Abbreviation | State Name |
| --- | --- | --- |
| 1 | AL | Alabama |
| 2 | AZ | Arizona |
| 3 | AR | Arkansas |
| 4 | CA | California |
| 5 | CO | Colorado |
| 6 | CT | Connecticut |
| 7 | DE | Delaware |
| 8 | FL | Florida |
| 9 | GA | Georgia |
| 10 | ID | Idaho |
| 11 | IL | Illinois |
| 12 | IN | Indiana |
| 13 | IA | Iowa |
| 14 | KS | Kansas |
| 15 | KY | Kentucky |
| 16 | LA | Louisiana |
| 17 | ME | Maine |
| 18 | MD | Maryland |
| 19 | MA | Massachusetts |
| 20 | MI | Michigan |
| 21 | MN | Minnesota |
| 22 | MS | Mississippi |
| 23 | MO | Missouri |
| 24 | MT | Montana |
| 25 | NE | Nebraska |
| 26 | NV | Nevada |
| 27 | NH | New Hampshire |
| 28 | NJ | New Jersey |
| 29 | NM | New Mexico |
| 30 | NY | New York |
| 31 | NC | North Carolina |
| 32 | ND | North Dakota |
| 33 | OH | Ohio |
| 34 | OK | Oklahoma |
| 35 | OR | Oregon |
| 36 | PA | Pennsylvania |
| 37 | RI | Rhode Island |
| 38 | SC | South Carolina |
| 39 | SD | South Dakota |
| 40 | TN | Tennessee |
| 41 | TX | Texas |
| 42 | UT | Utah |
| 43 | VT | Vermont |
| 44 | VA | Virginia |
| 45 | WA | Washington |
| 46 | DC | Washington D.C. |
| 47 | WV | West Virginia |
| 48 | WI | Wisconsin |
| 49 | WY | Wyoming |

Table 4 Coefficients of the linear mixed-effects model including state-level vaccination rate instead of political beliefs.

| Variable | Coefficient | Std. Error | P-value |
| --- | --- | --- | --- |
| *Intercept* | 0.624 | 0.086 | 0.000*** |
| *Topic:Preventive_Measures* | 0.091 | 0.020 | 0.000*** |
| *Topic:Quarantine* | 0.091 | 0.020 | 0.000*** |
| *Topic:Symptoms* | 0.005 | 0.020 | 0.819 |
| *Topic:Testing* | -0.095 | 0.020 | 0.000*** |
| *Topic:Vaccination* | -0.032 | 0.020 | 0.117 |
| *Topic:Virus* | 0.088 | 0.020 | 0.000*** |
| *Timeframe* | -0.009 | 0.029 | 0.745 |
| *Topic:Preventive_Measures* x *Timeframe* | -0.039 | 0.009 | 0.000*** |
| *Topic:Quarantine* x *Timeframe* | -0.048 | 0.009 | 0.000*** |
| *Topic:Symptoms* x *Timeframe* | 0.002 | 0.009 | 0.817 |
| *Topic:Testing* x *Timeframe* | -0.013 | 0.009 | 0.125 |
| *Topic:Vaccination* x *Timeframe* | 0.004 | 0.009 | 0.684 |
| *Topic:Virus* x *Timeframe* | 0.010 | 0.009 | 0.254 |
| *Vaccination_Rate* | -0.132 | 0.052 | 0.011** |
| *Topic:Preventive_Measures* x *Vaccination_Rate* | 0.197 | 0.054 | 0.000*** |
| *Topic:Quarantine* x *Vaccination_Rate* | 0.357 | 0.054 | 0.000*** |
| *Topic:Symptoms* x *Vaccination_Rate* | 0.057 | 0.054 | 0.298 |
| *Topic:Testing* x *Vaccination_Rate* | 0.193 | 0.054 | 0.000*** |
| *Topic:Vaccination* x *Vaccination_Rate* | 0.350 | 0.054 | 0.000*** |
| *Topic:Virus* x *Vaccination_Rate* | 0.101 | 0.054 | 0.062* |
| *Group Var* | 0.755 | 0.546 | 0.167 |
| x *interaction effect between variable x and y;* | *** p < 0.01; | ** p < 0.05; | * p < 0.1; |
|  |  |  |  |


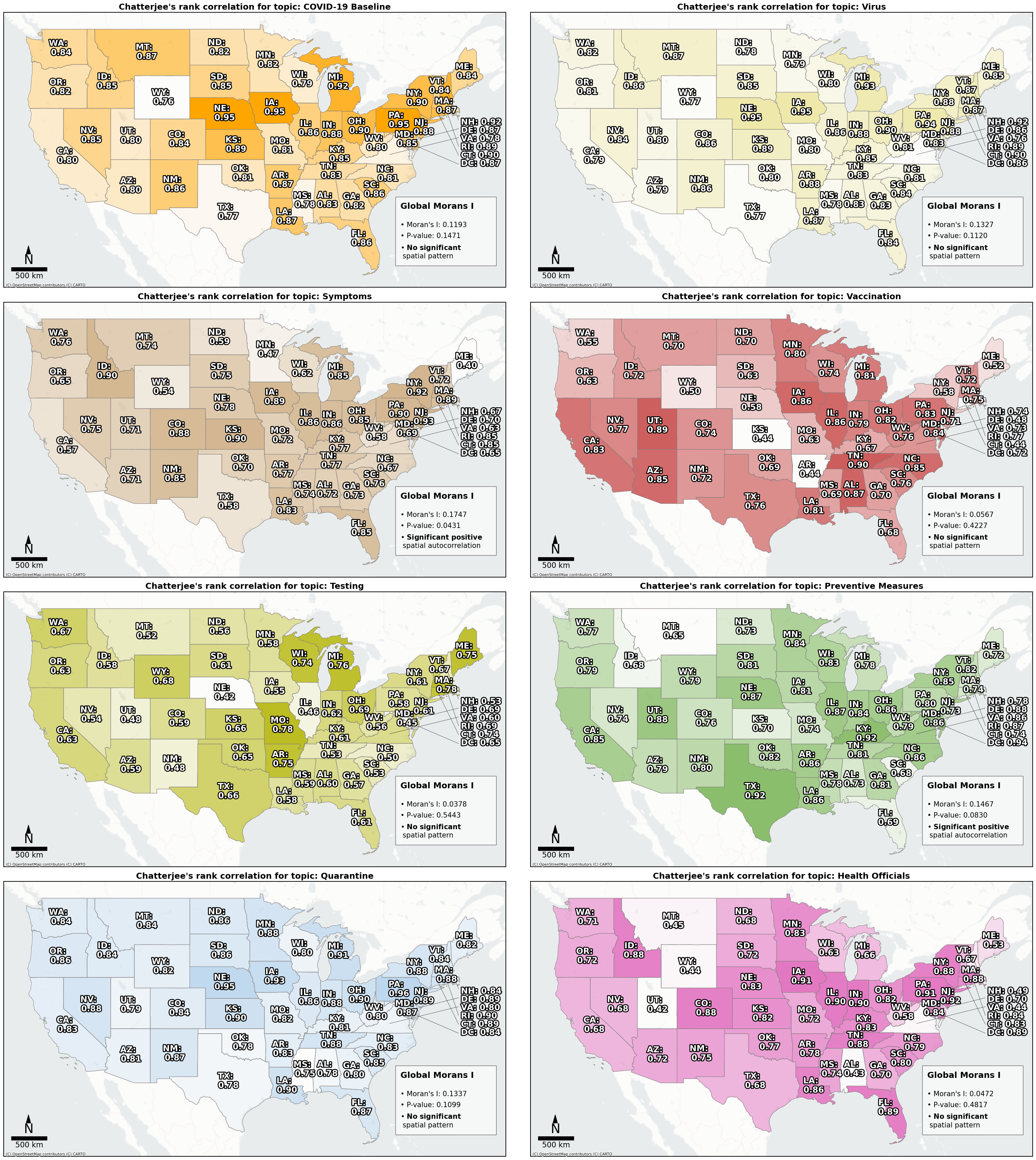


Figure 6 Chatterjee’s rank correlation for each geo-social media topic for mainland US states in timeframe 1.


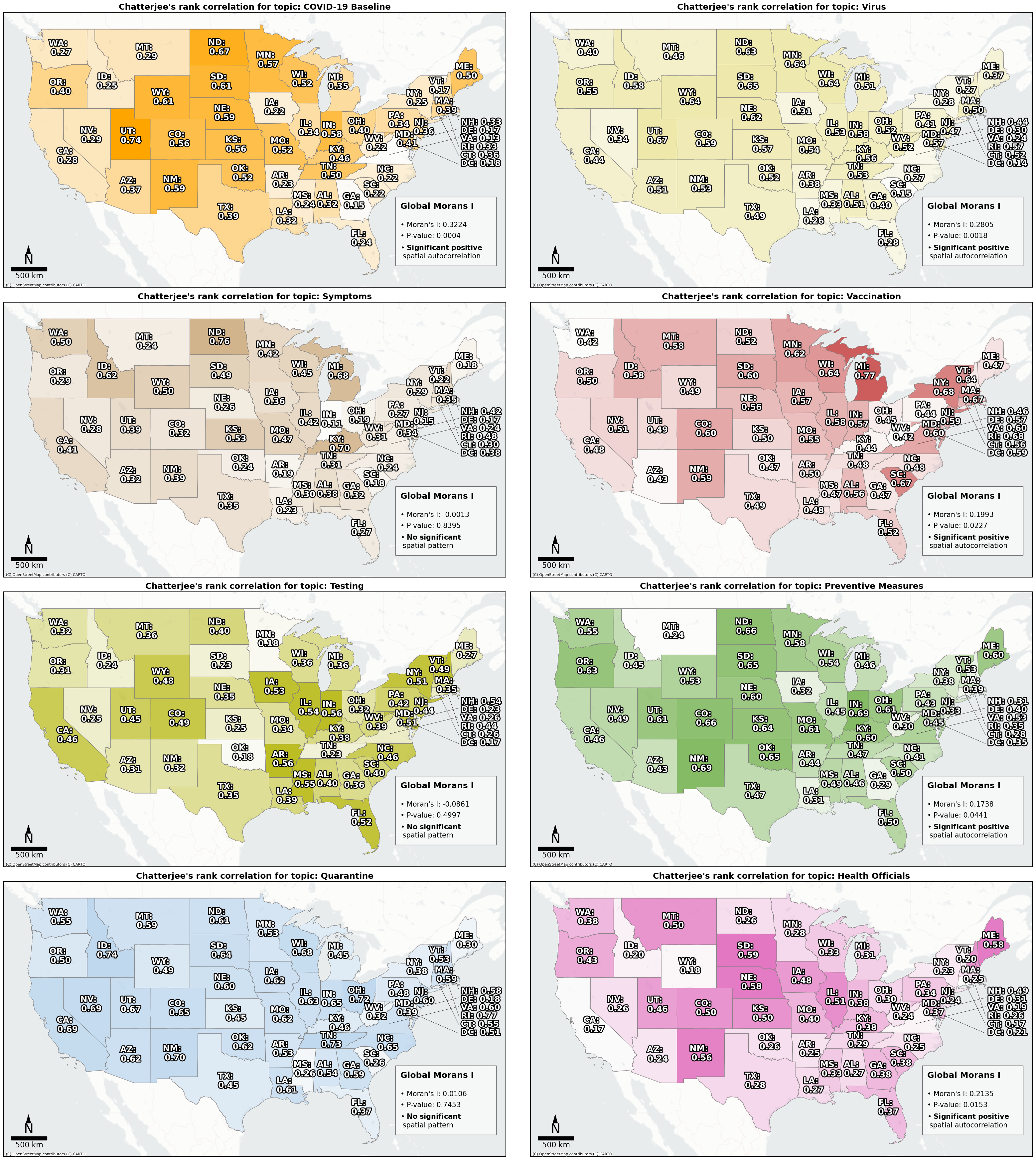


Figure 7 Chatterjee’s rank correlation for each geo-social media topic for mainland US states in timeframe 3.


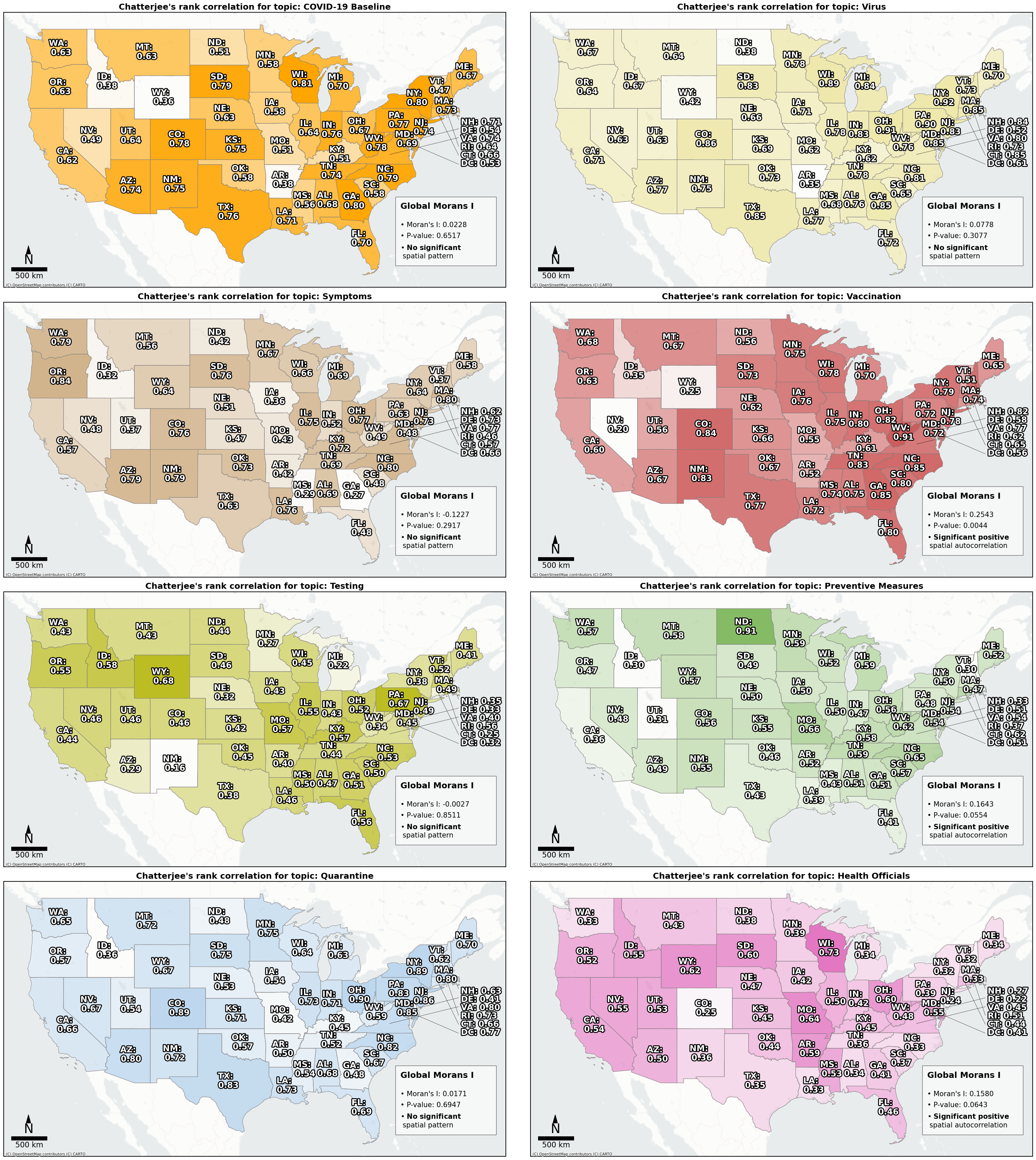


Figure 8 Chatterjee’s rank correlation for each geo-social media topic for mainland US states in timeframe 4.


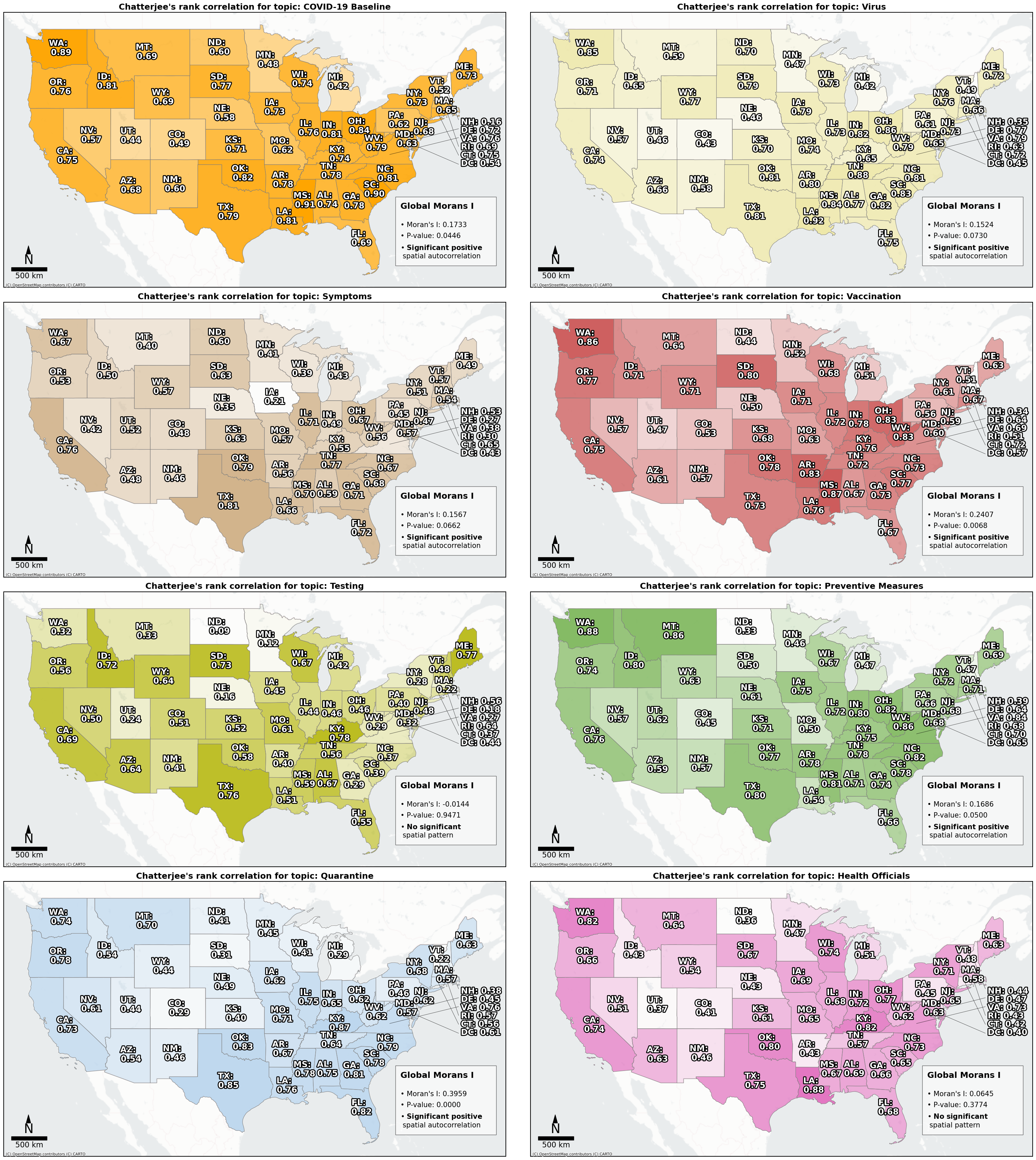


Figure 9 Chatterjee’s rank correlation for each geo-social media topic for mainland US states in timeframe 5.


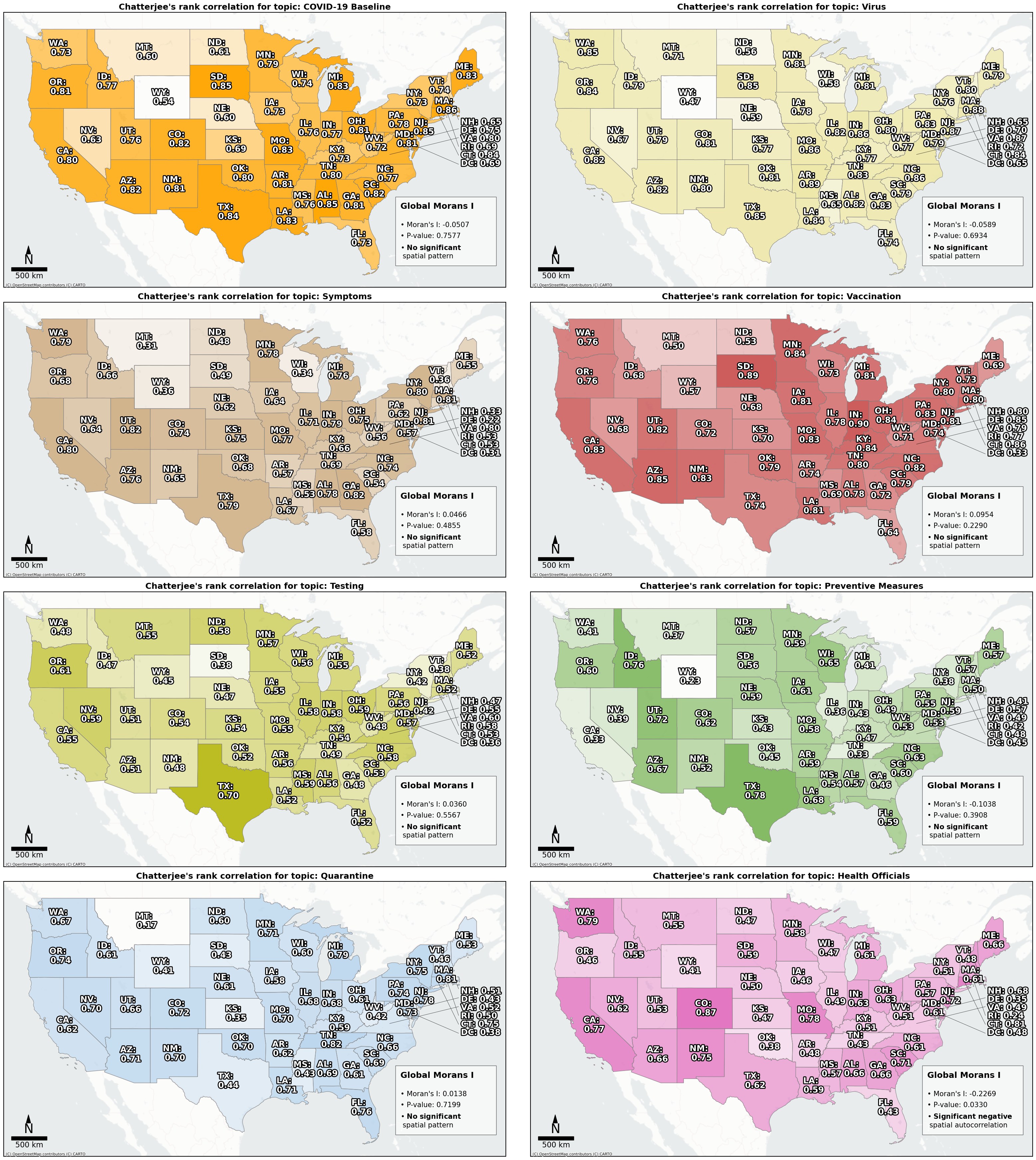


Figure 10 Chatterjee’s rank correlation for each geo-social media topic for mainland US states in timeframe 6.


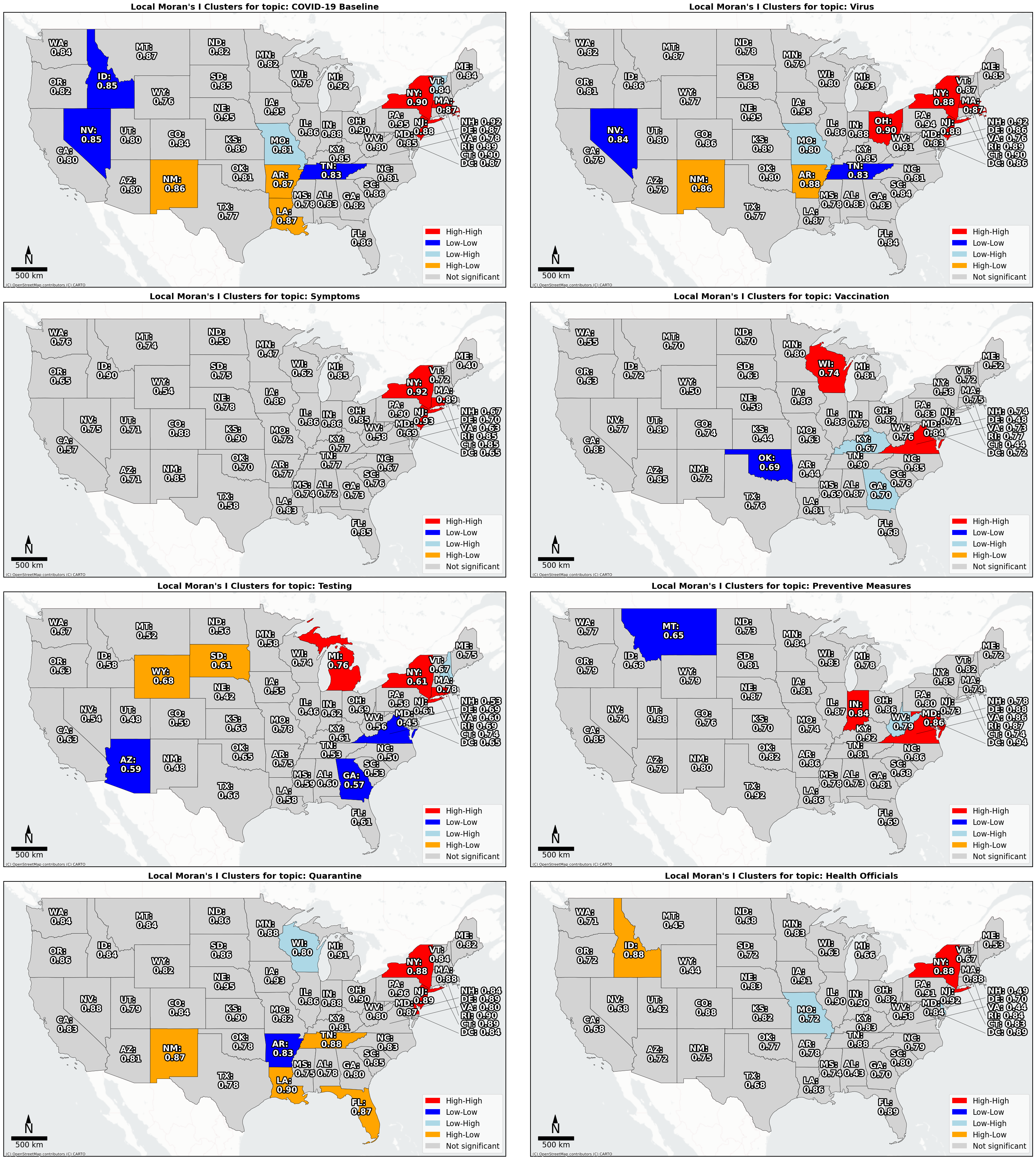


Figure 11 Local spatial autocorrelation over Chatterjee’s rank correlation for each geo-social media topic for mainland US states in timeframe 1.


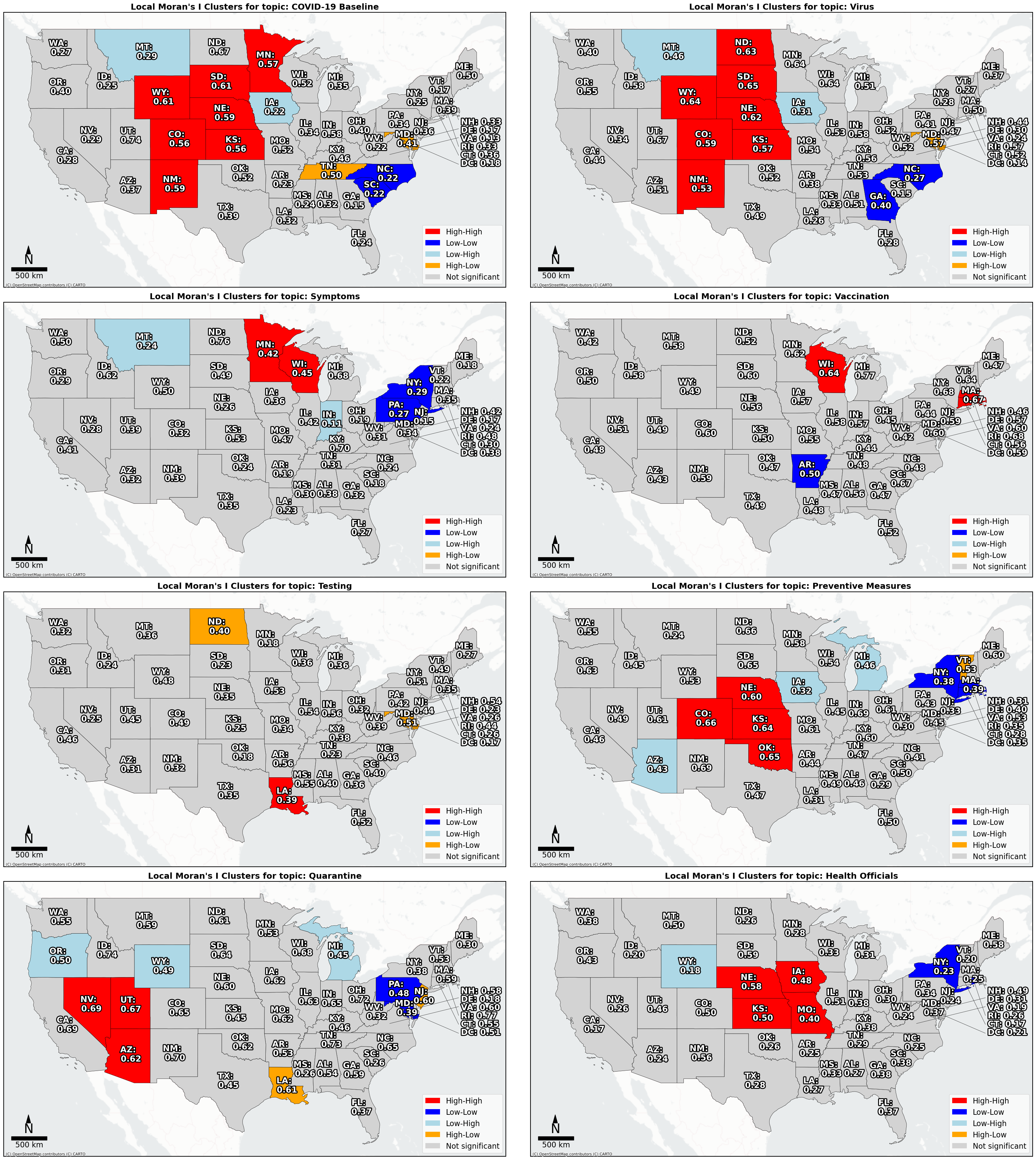


Figure 12 Local spatial autocorrelation over Chatterjee’s rank correlation for each geo-social media topic for mainland US states in timeframe 3.


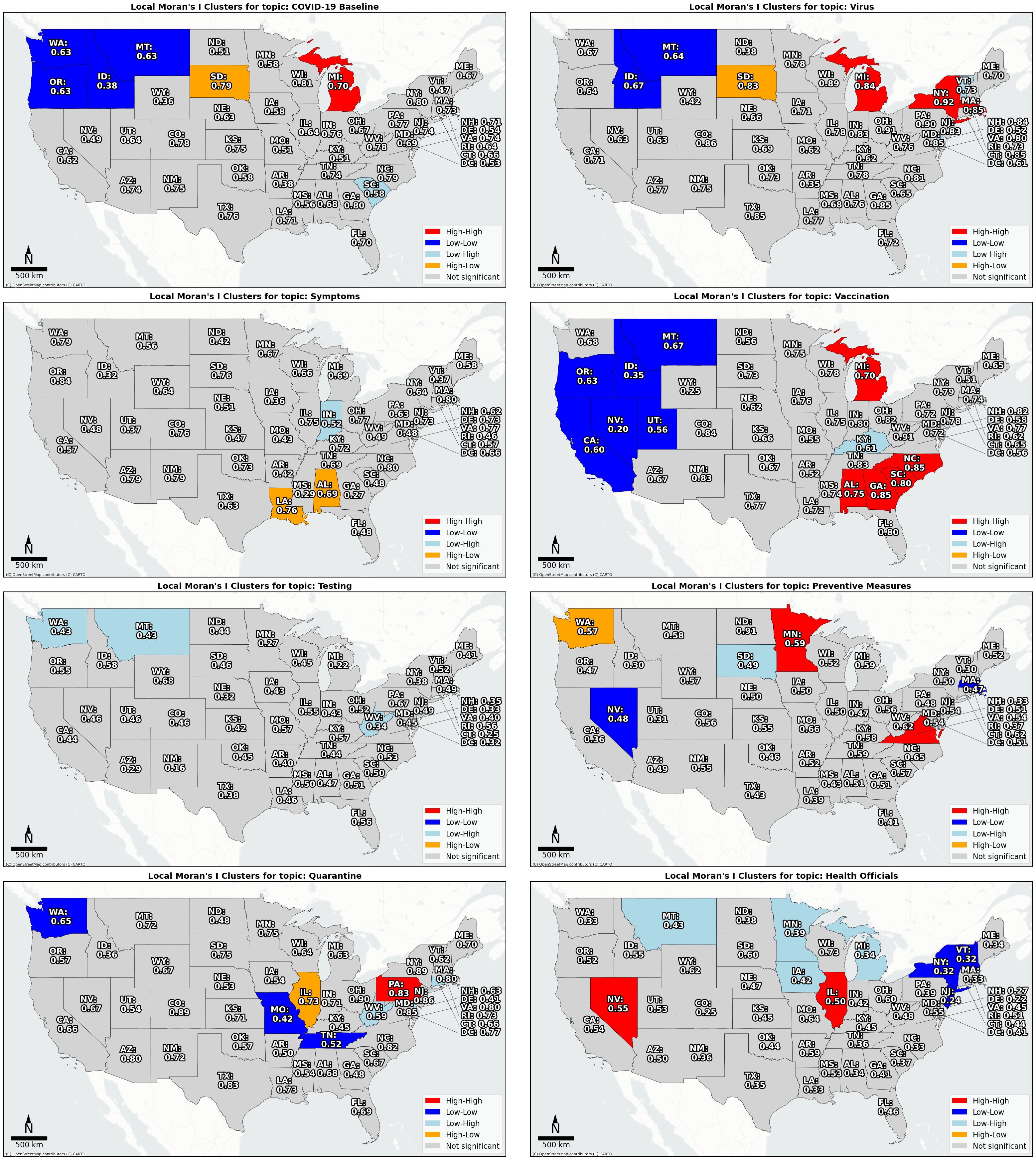


Figure 13 Local spatial autocorrelation over Chatterjee’s rank correlation for each geo-social media topic for mainland US states in timeframe 4.


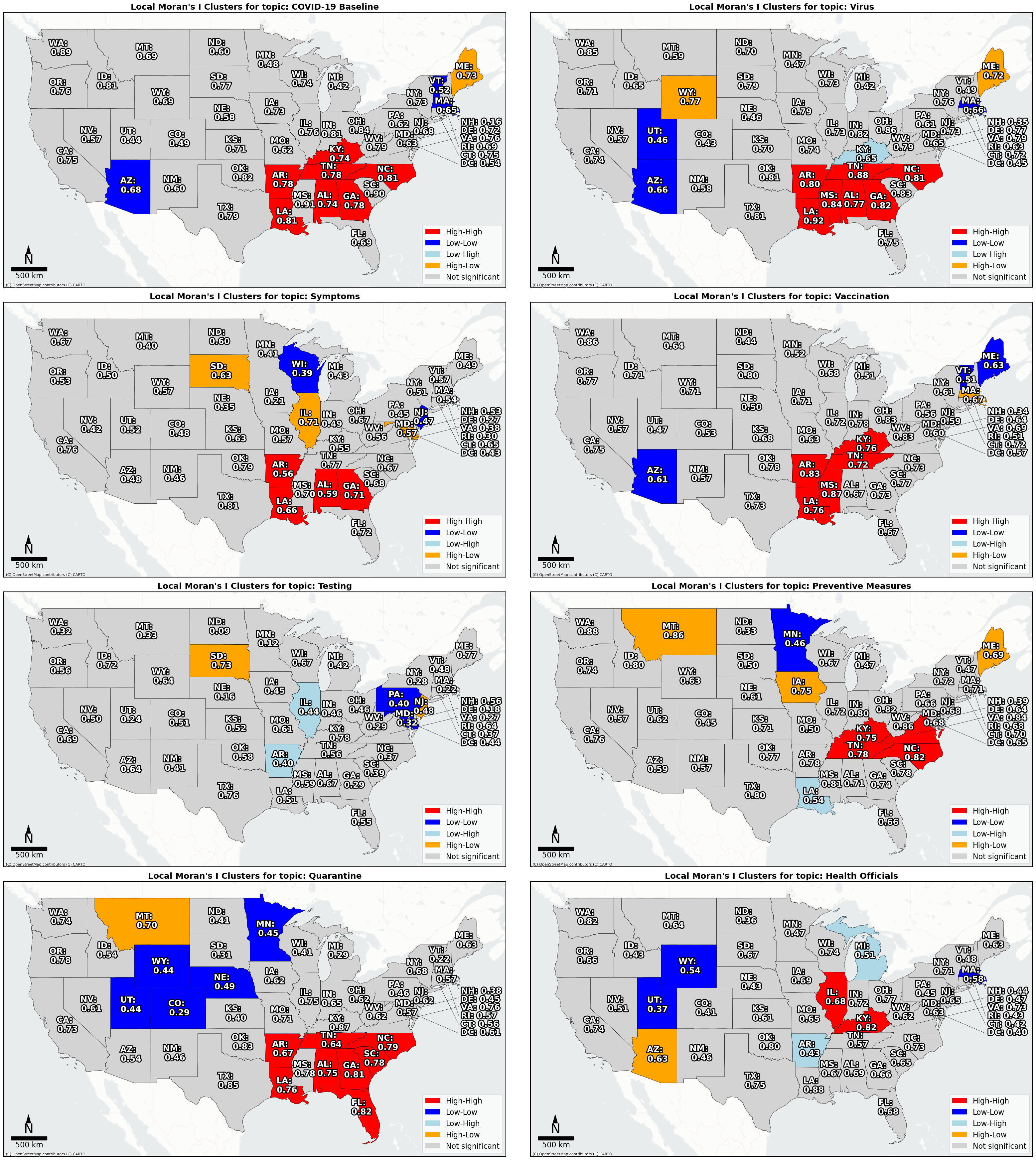


Figure 14 Local spatial autocorrelation over Chatterjee’s rank correlation for each geo-social media topic for mainland US states in timeframe 5.


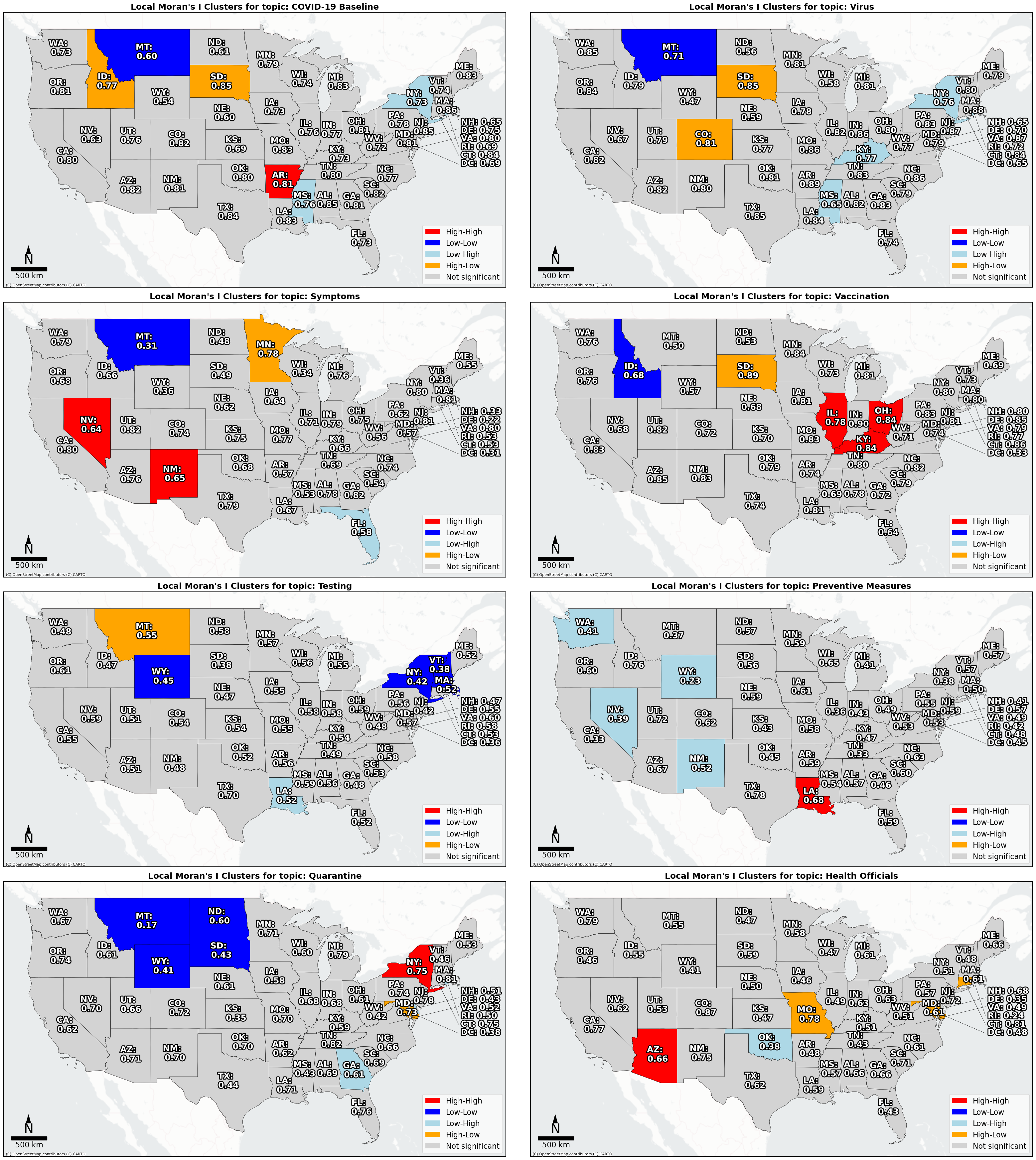


Figure 15 Local spatial autocorrelation over Chatterjee’s rank correlation for each geo-social media topic for mainland US states in timeframe 6.
